# Effects of Class C and Class F Fly Ash on Mechanical and Microstructural Behavior of Clay Soil—A Comparative Study

**DOI:** 10.3390/ma15051845

**Published:** 2022-03-01

**Authors:** Canan Turan, Akbar A. Javadi, Raffaele Vinai

**Affiliations:** Department of Engineering, University of Exeter, Exeter EX4 4QF, UK; a.a.javadi@exeter.ac.uk (A.A.J.); r.vinai@exeter.ac.uk (R.V.)

**Keywords:** class C fly ash, class F fly ash, clay, triaxial test, consolidation test, UCS test, SEM

## Abstract

A large amount of coal fly ash produced in thermal power plants is disposed of in landfills which causes many environmental problems. The utilization of fly ash can be encouraged in geotechnical engineering projects. In this paper, the effects of class C and class F fly ash on the mechanical and microstructural behavior and stabilization of clay soil were evaluated through a program of laboratory experiments. The experiments included compaction, unconfined compressive strength, consolidated-undrained triaxial, one-dimensional consolidation tests, and scanning electron microscopy analysis on samples of fly ash-stabilized clay soil after 1, 7, and 28 days of curing. The tests were conducted on mixtures of clay with class C or class F fly ash, ranging from 0% to 30% of the soil. Experimental results showed that the strength parameters and permeability of the stabilized soil improved while the compression and swelling indices decreased by the addition of fly ash and by the increase of curing days. The results obtained from the mechanical tests agreed with the results from the SEM analysis. Based on the results, the soil could be successfully stabilized by using class C fly ash. The improvements in strength, swelling, and permeability parameters of the stabilized soil were higher with the class C fly ash compared with class F fly ash.

## 1. Introduction

Fly ash is an industrial by-product generated during the combustion of coal in thermal power plants [1,2]. It is generated in large amounts in many countries [3]. Over 65% of the produced fly ash is disposed of in landfills [4]. If the fly ash, as a waste material, is not managed well, it can lead to serious environmental and health problems [4,5]. However, many characteristics of fly ash such as low compressibility, high shear resistance, high strength and pozzolanic characteristics offer it an important role in improving the properties of soil in geotechnical applications [2,6]. The stabilization of soft soils with the addition of fly ash not only addresses the environmental issues of disposal of fly ash but can also provide technological solutions for soil improvement [2].

Therefore, the stabilization of different types of soil with fly ash has encouraged various researchers to carry out experimental or/and field studies. The available literature on clay soils stabilized with fly ash is summarized in Table 1. These investigations have generally pointed out that the inclusion of fly ash can improve the soil structure and characteristics in many aspects, including strength, stiffness, permeability, and compressibility.

Seyrek [17] conducted Atterberg limits, compaction, swelling potential, swelling pressure tests, and unconfined compressive strength (UCS) tests to analyze the effects of class C and class F fly ash on both high plasticity clay (CH) and low plasticity clay (CL) at 1, 7, and 28 days of curing periods. They showed that the plasticity index of both types of fly ash stabilized soil decreased with the addition of 20% fly ash. However, beyond 20% fly ash content, an increase in plasticity index was observed. The results from the compaction tests showed that the maximum dry unit weight of the soil decreased, and optimum moisture content increased with the addition of fly ash. The amount of swelling and swelling pressure of the soil decreased significantly by increasing the fly ash content. However, the changes became insignificant beyond 25% class C fly ash. For the CH soil, adding 30% class F fly ash gave similar results in terms of reduction in swelling compared to 10% class C fly ash. The peak UCS values for the samples with 28 days of curing were found to be 657 kPa and 915.5 kPa with 25% class F fly ash and 30% class C fly ash, respectively. It was concluded that class C fly ash provided remarkable improvement in compressive strength with increasing the curing. Likewise, Phani Kumar and Sharma [10] assessed plasticity, strength, swelling, and compaction characteristics of clayey soil with the addition of different percentages of low calcium fly ash. Based on their results, the plasticity index and swelling characteristics of the stabilized soil decreased by approximately 50% with 20% fly ash inclusion. On the other hand, the swelling potential was insignificant after the addition of 20% fly ash. Undrained shear strength increased by about 27% with 20% of fly ash inclusion. According to compaction test results, the optimum moisture content decreased by about 25%, and the maximum dry unit weight increased by about 5% with 20% of fly ash inclusion. In a follow-up study, Phanikumar and Sharma [13] investigated the effects of class C fly ash using oedometer tests and the cylindrical jar method to determine free swell index, swell potential, swelling pressure, and compression index for an expansive and a nonexpansive high plasticity clay. The plasticity indices of the expansive and the nonexpansive clay were found to be 131–53 and 29, respectively. Fly ash contents up to 20% (based on dry weight of the soil) were added to the soil samples. It was found that the free swell index decreased by approximately 50% for the expansive clay with the addition of 20% fly ash based on the cylindrical jar method. The compression index significantly decreased with the addition of fly ash on both expansive and nonexpansive soils. On the other hand, the effect of fly ash in improving the compressibility properties of the expansive clay was greater compared to the nonexpansive clay. Cokca [7] assessed the effects of four stabilization materials (high calcium and low calcium fly ash, lime, and cement) on the swelling potential of a clay soil. The amounts of lime and cement used were between 0–8%, while the amounts of fly ash used were between 0–25%. Based on the results, the classification of the stabilized soil changed from CH to CL, MH-ML, ML, and CL with the addition of 8% lime, 8% cement, 25% high calcium fly ash, and 25% low calcium fly ash, respectively. The swelling potential of all the samples stabilized with fly ash, cement, or lime decreased significantly. The highest reduction in swelling potential was 68% for low calcium fly ash. Jose et al. [18] also investigated the effects of class F fly ash on an expansive soil. The artificial soil (natural soil with bentonite) was used which had a liquid limit of 62%. They reported that the value of free swell index decreased from about 71% to 39% by adding 15% of fly ash. Moreover, the liquid limit of the soil decreased by about 36%, and the compressive strength increased by about 43% with the addition of 15% fly ash.

Prabakar et al. [8] carried out a number of tests to evaluate compaction, shear strength, California bearing ratio (CBR), and swelling characteristics of soils stabilized with the addition of fly ash ranging from 9 to 46%. They considered three different types of soils: CL (inorganic clay with low plasticity), OL (organic soil with low plasticity), and MH (inorganic silt with high plasticity). The results showed that the dry density of all soil types decreased between 15% and 20% by adding the fly ash. The shear strength of all the soil types increased nonlinearly with the fly ash content. The swelling potential of the soil also decreased, and the CBR value of the soil improved with the addition of fly ash. Sezer et al. [9] studied the effects of high lime fly ash with different percentages and curing times by applying compaction, UCS, and shear strength tests on a high plasticity clay soil. The fly ash contents used were 0, 5, 10, and 15% of dry weight of the soil, and the curing times were 1, 7, 28, and 90 days. They reported that the maximum dry density decreased, while the optimum moisture content increased with the addition of fly ash. The unconfined compressive strength, cohesion, and friction angle improved with the addition of fly ash. According to the UCS results, the strength parameters of the soil improved inconsiderably after 28 days. The optimal amount of fly ash to stabilize the soil was found to be 15%. Senol et al. [11] conducted compaction, UCS, and CBR tests on class C fly ash stabilized clay soil to investigate the suitability in pavement design. Tests were conducted after seven days of curing in normal room temperature on mixtures of the soil with between 10–20% class C fly ash. It was concluded that the inclusion of fly ash provides a significant improvement in the UCS and CBR values. Therefore, it was highly recommended that fly ash stabilized soil can be used as soft subgrade material in the field. Brooks [15] investigated the effects of class C fly ash and rice husk ash on clay soil by conducting UCS, CBR, compaction, and swell-shrinkage tests. According to the UCS test results, failure stress increased approximately 106% with 25% fly ash content. However, beyond 25%, the failure stress decreased with further increase in the fly ash content. The swelling potential of the stabilized soil decreased, and 25% was recommended as the optimum fly ash content for stabilization of the clay soil. Bin-Shafique et al. [14] carried out cyclic wetting-drying and freeze-thaw tests to study the long-term performance of low- and high- plasticity clay soils stabilized with class C fly ash. They carried out UCS, plasticity index, and vertical swell tests on soil samples stabilized with the fly ash ranging from 0–20%. It was shown that the UCS of both types of soil improved by a factor of four, and the swelling potential and plasticity decreased about 75% and 50%, respectively, with 20% fly ash content. The wetting-drying cycles with saline water or tap water did not have an impact on strength parameters, swelling potential, and plasticity. However, strength parameters decreased about 40% for CH and 20% for CL after freeze-thaw cycles. Nevertheless, for both soil types, after the freeze-thaw cycles the fly ash-stabilized soil still had a significantly greater strength than the unstabilized soil (control sample).

Edil et al. [12] analyzed the effects of class C fly ash on six inorganic soils (CL and CH) and one organic soil (OH) by applying CBR and resilient modulus (M_r_) tests. They reported, on the basis of the test results, that the inclusion of fly ash (10–30%) provides a significant increase in the CBR and M_r_ parameters of the inorganic soils. The CBR values increased four and eight times with the addition of 10% and 18% fly ash, respectively. On the other hand, the effect of fly ash on organic soil was insignificant. Tastan et al. [16] investigated the effects of fly ash in stabilization of an organic soil. Resilient modulus (M_r_) and UCS tests were carried out on soil stabilized with 10%, 20%, and 30% fly ash. The results indicated that the different fly ash contents and soil types significantly affected the effectiveness of the stabilization. For example, the UCS increased about 400 kPa with addition of fly ash to two types of clayey soil with organic content less than 10%. However, by adding the same amount of fly ash, the UCS of an organic sandy silty peat with 27% organic content only increased 100 kPa. 

The review of the literature suggests that fly ash stabilization of soil has great potential for improving the mechanical and physical properties of geomaterials. Common tests used for the study of fly ash stabilized soils are UCS, free swell index, consistency limits, and CBR tests. However, little information is available on shear and consolidation behavior of fly ash stabilized clay soils. Furthermore, previous studies did not fully clarify the different effects of using class C fly ash and class F fly ash on shear, consolidation, and microstructural behavior of stabilized soils. Therefore, there is a need to improve the fundamental understanding of how class C and class F fly ash affect the overall shear, consolidation, and microstructural behavior on soil samples. Shear strength parameters play an important role in estimating the bearing capacity of soils and in the assessment of the stability of geotechnical structures, while consolidation parameters allow the analysis of settlement behavior of soils.

This study presents a comparison of class C and class F fly ash on the stabilization of a clay soil (kaolinite). Their effects are evaluated through a program of laboratory tests including compaction, UCS, consolidated-undrained (CU) triaxial, one-dimensional consolidation (oedometer) tests, and scanning electron microscopy (SEM) analysis. The outcome of the study will improve the current understanding of the effects of class C and class F fly ash on the mechanical behavior and microstructural characteristics of stabilized soils, help determine and compare the suitability of class C and class F fly ash as alternative soil stabilizing agents and encourage the utilization of fly ash to reduce the environmental impacts of fly ash disposal. Another focus will be to identify the optimal amounts of the two types of fly ash for the stabilization of clay soil.

## 2. Materials Characterization

### 2.1. Soil

A kaolinite powder (China clay) was used in this study. Atterberg limit, specific gravity, and standard proctor tests were conducted to characterize the soil, according to the British Standards (BS) [19,20]. The soil was found to have a liquid limit of 49%, plastic limit of 25%, and plasticity index of 24%. According to the plasticity chart based on the British system [21], the soil was classified as clay with intermediate plasticity (CI). The compaction characteristics of the soil were found from the standard proctor test. The soil had a maximum dry unit weight (ɣ_dmax_) of 15.2 kN/m^3^ and optimum moisture content (w_opt_) of 21%. The specific gravity of the soil was found to be 2.6 from the small pycnometer test.

**Figure 1:**
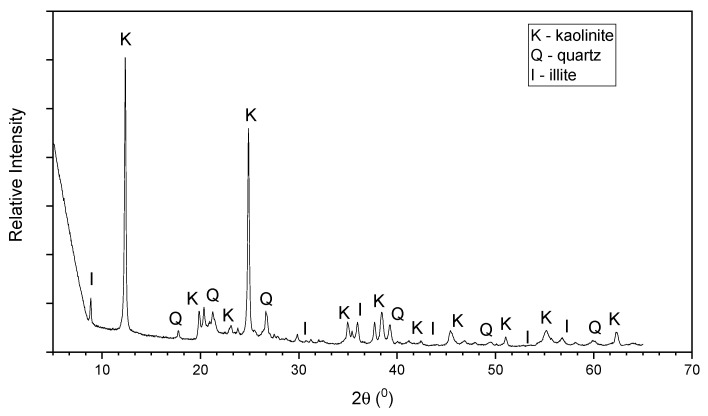
X-ray diffraction (XRD) pattern of the clay.

**Figure 2:**
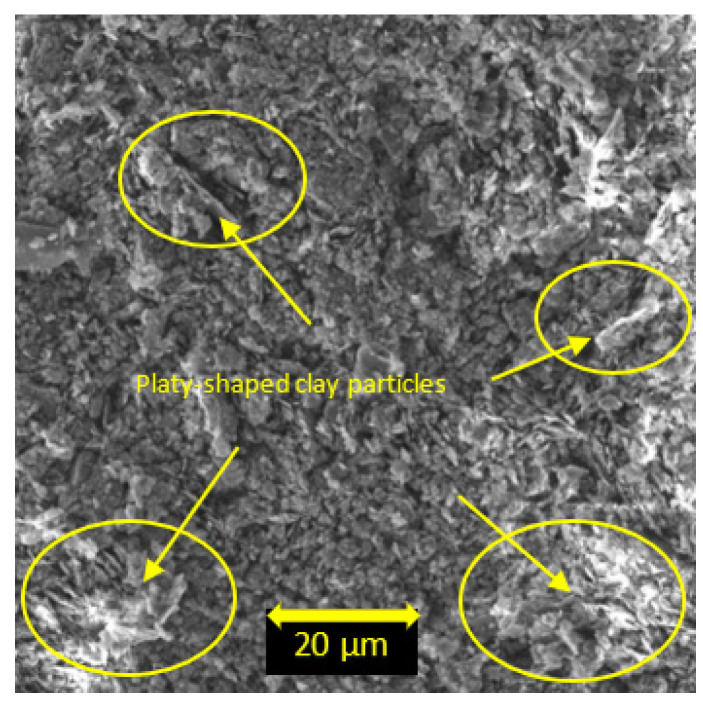
Scanning Electron Microscopy (SEM) image of the soil.

X-ray diffraction (XRD) analysis was carried out to obtain mineralogical characterization of the soil using a Bruker D8 advanced XRD equipment (Exeter, UK). The recorded angular range was 5 to 65° (2θ) from the X-Ray generator. SEM analysis was conducted with 2.00 kx magnification factor on the soil, with the use of TESCAN VEGA3 SEM detector (Exeter, UK). Based on the XRD results, the dominant minerals of the soil were found to be kaolinite, quartz, and illite (Figure 1). Also, according to the SEM analysis, the soil showed plate-like fragments with different thicknesses (Figure 2).

### 2.2. Fly Ashes

Class C fly ash was sourced from the MUEG company in Germany (Braunsbedra), and class F fly ash was supplied from Power Minerals Ltd. in the UK (Birmingham). Both fly ashes were silt sized. Thus, the parameters of the plasticity index could not be evaluated, and it was recorded as nonplastic (NP). The values of specific gravity of the class C and the class F fly ash were found to be 2.4 and 2.32, respectively.

**Figure 3:**
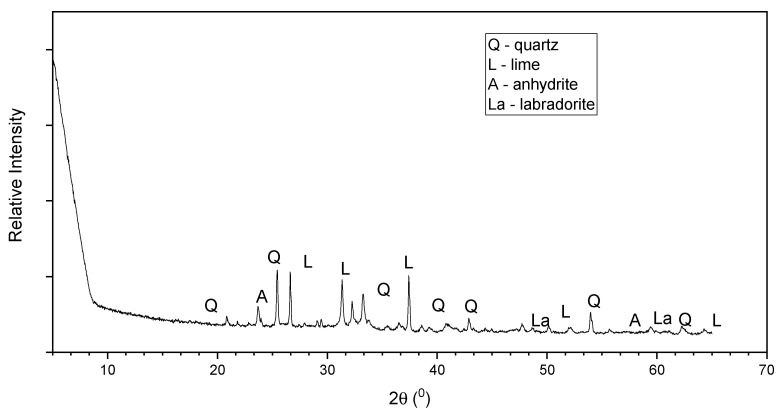
X-ray diffraction (XRD) pattern of class C fly ash.

**Figure 4:**
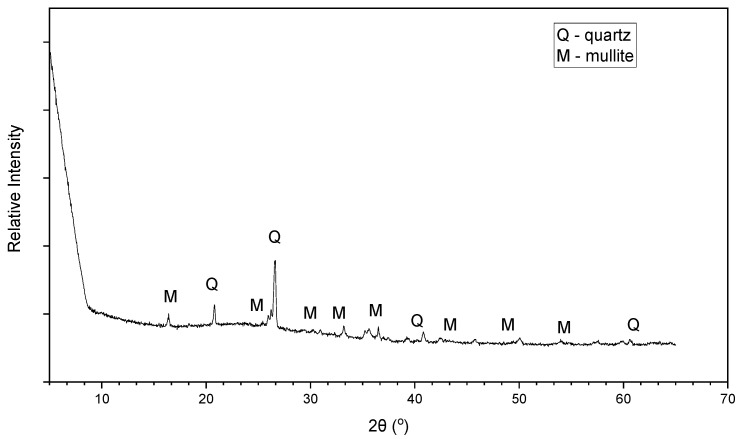
X-ray diffraction (XRD) pattern of class F fly ash.

**Figure 5:**
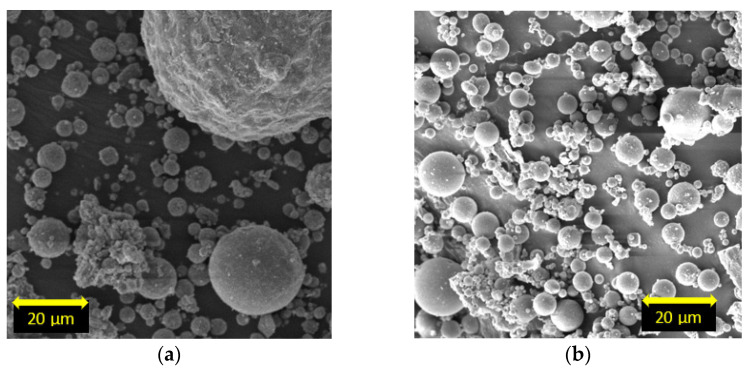
SEM images of (**a**) class C and (**b**) class F fly ash.

The average chemical compositions of the class C and the class F fly ash are shown in Table 2. The fly ashes were classified based on the ASTM C618 [22] standard. The mineralogical compositions of the class C and class F fly ash are presented in Figure 3 and Figure 4, respectively, based on the XRD analysis. It is seen that the main crystalline phases of the class C fly ash include lime, quartz, anhydride, and labradorite, while the class F fly ash includes quartz and mullite. SEM analysis was conducted on both fly ashes, and the morphology of both fly ash types was generally found to be fine and spherical (Figure 5a,b).

## 3. Sample Preparation

Control samples and fly ash stabilized soil samples were prepared by static compaction using an Instron 3382 Floor Model Universal Loading System (Exeter, UK) for the UCS, triaxial, and one-dimensional consolidation tests. Each layer was compacted by increasing the load at a displacement rate of 2.5 mm/min up to a constant maximum load. The maximum dry unit weight of the compacted samples was recorded between 13.9–15.2 kN/m^3^ based on the different fly ash contents of the stabilized soil samples. The relevant optimum moisture contents of the control sample and fly ash-stabilized soil samples obtained from standard proctor tests were used. The prepared samples were 50 mm in diameter and 100 mm in height and were compacted in 3 layers for UCS and CU triaxial tests. The samples, 50 mm in diameter and 20 mm in height, were prepared in one layer for one-dimensional consolidation tests. After compaction, the samples were sealed and stored in laboratory conditions (23–26 °C) and cured in vacuum desiccators for 1, 7, and 28 days before testing.

## 4. Experimental Program

The experiments conducted in this study focused on the effects of the type and amount of fly ash on the mechanical and microstructural behavior of soil. A series of laboratory tests including compaction, UCS, CU triaxial, one-dimensional consolidation tests, and SEM were conducted on the control (neat clay) and stabilized soil samples. 

### 4.1. Compaction Tests

To finds the optimum moisture content and maximum the dry unit weight of the control sample and the soil samples stabilized with 5%, 10%, 15%, 20%, 25%, and 30% fly ash (based on weight of the dry soil), standard proctor compaction tests were conducted according to BS [20]. These percentages were particularly selected based on the recommendations from the literature. [7,9,10,13,14,15,17]. The samples were compacted in three layers in a 1 L mold, and 27 blows were applied for each layer using a 2.5 kg rammer. After the initial mixing of the samples, the compaction was applied without any delay. This is because, ɣ_dmax_ might be affected with compaction delays. Mahvash et al. [23] stated that due to the quick hydration reaction of fly ash, it can be bonded with the soil particles in a loose state, and these bonds could cause disruption of material during the compaction process.

### 4.2. Unconfined Compressive Strength Tests

UCS tests were conducted on the control samples and samples of soil stabilized with 5%, 10%, 15%, 20%, 25%, and 30% fly ash with 1-, 7-, and 28-day curing times. The samples were loaded between two metal plates at a displacement rate of 1 mm/min. All UCS tests were conducted on duplicate samples and average results were used. 

### 4.3. Consolidated-Undrained Triaxial Tests

Based on the results obtained from the UCS tests, the fly ash contents of 15% and 25% were chosen for the triaxial tests. According to the UCS tests, the strength parameters generally decreased after 25% of fly ash content. Therefore, 25% fly ash was considered as optimal. A sample of 15% fly ash was selected to compare and determine the effects of changes in fly ash content from 15% to 25%. Cylindrical samples were prepared by static compaction. Isotropically consolidated undrained triaxial tests were conducted according to the BS [24]. The main phases of the tests included saturation, consolidation, and shearing. GDS triaxial testing equipment (GDS instruments, London, UK) and GDS software (GDSlab v2.8.2.4, London, UK) were used to conduct the triaxial experiments at confining pressures of 200, 400, and 600 kPa. The results were analyzed in terms of stress-strain behavior, and shear strength and critical state parameters of control samples and soil samples stabilized with 15% and 25% of fly ash with 1-, 7-, and 28-day curing times. 

### 4.4. One-Dimensional Consolidation Tests

A series of standard one-dimensional consolidation tests were carried out to determine the consolidation properties of the control sample and the fly ash-stabilized soil with 15% and 25% fly ash. The tests were conducted in accordance with the British Standard, BS [25]. The curing times of the samples were 1, 7, or 28 days for both fly ash types. According to the procedures of the standard, in each loading step the stress was doubled, at least four incremental steps were applied on all samples, and loading was done in a smaller number of unloading steps. The samples were sequenced to apply vertical stresses of 10, 20, 40, and 80 kPa (loading stages), 40 and 10 kPa (unloading stages). Each loading or unloading step of the consolidation tests was kept for 24 h. 

### 4.5. Scanning Electron Microscopy

The microstructural investigation of the control sample and the soil samples stabilized with class C and class F fly ash was carried out using TESCAN Vega 3 SEM. SEM tests were applied on the stabilized soil samples with 1, 7, and 28 days of curing time. The accelerating voltage of 10 kV and beam intensity of 10 were used in this study. The dried samples were mounted on the stubs using carbon tape and coated by Quorum Q150 TES Sputter (Exeter, UK) coater to improve their conductivity prior to the SEM analysis. 

## 5. Results and Discussion

### 5.1. Compaction Tests

**Figure 6:**
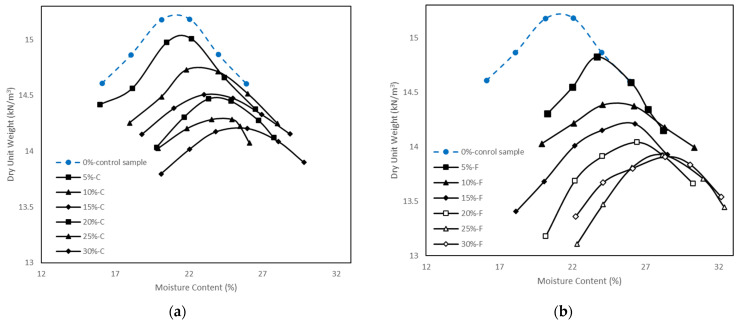
Compaction curves for control sample and soil samples stabilized with (**a**) class C fly ash and (**b**) class F fly ash.

The compaction curves of the control sample and the soil samples stabilized with class C and class F fly ash are shown in Figure 6a,b. The results show that γ_dmax_ decreased and w_opt_ increased with the increase of the class C or class F fly ash content. This trend is similar to that found by other researchers [8,11,17,26,27,28,29]. The decrease in γ_dmax_ with the addition of fly ash is due to the lower specific gravity of fly ash than kaolinite [29,30,31]. The specific gravities of class C and class F fly ash in this study were 2.4 and 2.32, respectively. Therefore, the decrease of γ_dmax_ was smaller for the soil samples stabilized with class C fly ash compared to class F fly ash. Agglomeration and flocculation occurred due to the cation exchange reaction which resulted in a change of gradation in the stabilized soil samples. This could be another reason for the decrease in γ_dmax_ [3,32]. Seyrek [17] also argued that the immediate formation of cementitious products could decrease the value of γ_dmax_ in stabilized soil. According to Mir and Sridharan [27], the increase in w_opt_ with the addition of fly ash is due to the presence of some large and hollow spheres in fly ash situated in stabilized soil.

It is seen from Figure 6a,b that by increasing the fly ash content, the compaction curves shift to the bottom right of the graph. For example, the w_opt_ and γ_dmax_ changed from 21% and 15.2 kN/m^3^ for kaolinite to 25.8% and 14.2 kN/m^3^ with 30% of class C fly ash, and to 28.2% and 13.9 kN/m^3^ with 30% of class F fly ash addition. Also, the compaction curves for the stabilized soil with 15%, 20%, and 25% class C fly ash showed shallower peak in comparison with kaolinite. The shallow curve indicates that the soil samples stabilized with 15%, 20%, and 25% class C fly ash did not show significant change over a wide range of water content. However, this trend (shallow compaction curves) was not observed for the soil stabilized with class F fly ash. Hence, it can be said that soils stabilized with class C fly ash are more adaptable in field applications compared to class F fly ash stabilized soils.

### 5.2. Unconfined Compressive Strength Tests

**Figure 7:**
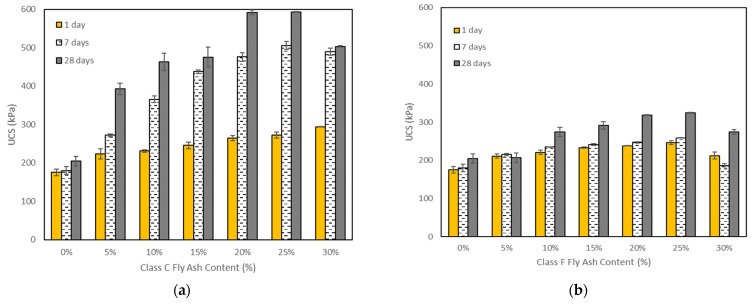
Effects of (**a**) class C and (**b**) class F fly ash contents on unconfined compressive strength with 1 day, 7 days, and 28 days of curing.

The relationship between the unconfined compressive strength (q_u_) and the fly ash content for different curing periods is presented in Figure 7a,b. The test results clearly demonstrate that the compressive strength increased with increasing the curing time for both types of fly ash. However, the effect of class C fly ash on the strength of the soil with 7 and 28 days of curing was much higher in comparison with class F fly ash. A similar trend was reported by Seyrek [17]. It is generally accepted that the higher the CaO/SiO_2_ ratio in fly ash, the greater the unconfined compressive strength and resilient modulus [16]. The class C fly ash used in the present study had a higher CaO/SiO_2_ ratio (32.4%/28.3%) and hence gave a higher compressive strength than the class F fly ash with ratio of 2.2%/46.8%. Furthermore, mineralogical analysis confirmed a non-negligible presence of lime, which has self-cementing properties, in the class C fly ash. Also, anhydrite in class C fly ash reacts with water and produces gypsum which has binding effects. On the other hand, class F fly ash does not have enough cementitious properties because of the low calcium content, and thus, it does not give a strong reaction with soil [3].

For both fly ash types and all curing times, the maximum value of unconfined compressive strength was found with 25% fly ash, and it showed a decrease from 25% to 30% of fly ash, except for mixtures with class C fly ash at one day of curing. These results are consistent with those reported by Dahale et al. [3] and Seyrek [17]. The unstabilized (control) soil samples had maximum axial stresses of 175 kPa, 180 kPa, and 204 kPa at 1, 7, and 28 days of curing, respectively. For the soils stabilized with class C fly ash, the peak stresses were 294 kPa with 30% fly ash at one day of curing, 506 kPa with 25% fly ash at seven days of curing, and 593 kPa with 25% fly ash at 28 days of curing. For the soils stabilized with 25% class F fly ash, the peak stresses were found to be 246 kPa at one day of curing, 259 kPa at seven days of curing, and 325 kPa at 28 days of curing. When the class C fly ash content was increased from 25% to 30%, the maximum axial stresses decreased from 506 kPa to 490 kPa at seven days of curing and from 593 kPa to 503 kPa at 28 days of curing. For the same increase in the fly ash content (from 25% to 30%), the maximum axial stresses of the soil stabilized with class F fly ash were decreased from 246 kPa to 212 kPa at one day of curing, from 259 kPa to 187 kPa at seven days of curing, and from 325 kPa to 274 kPa at 28 days of curing.

It can be concluded that class C fly ash was more effective in improving the compressive strength of the soil than class F fly ash. In addition, the curing time is an effective parameter in improving the strength and behavior of stabilized soil with class C and class F fly ash owing to their pozzolanic characteristics.

The values of elastic modulus (E) for the soils stabilized with class C and class F fly ash, obtained from the results of the UCS tests at 1, 7 and 28 days of curing are presented in Table 3. The elastic modulus was determined as the slope a tangent line of the linear part of the stress-strain curve [33]. The results indicate that, in general, for both types of fly ash, the elastic modulus increased with increasing the fly ash content up to 25%, beyond which, further increases in fly ash content resulted in a decrease in elastic modulus. However, the effect of class C fly ash in increasing the elastic modulus of the stabilized soil was greater than class F fly ash. It can be deduced that the improvement of the stiffness of the soil stabilized with class C fly ash is significant.

### 5.3. Triaxial Tests

#### 5.3.1. Effects of Fly Ash Content on the Stress-Strain Behavior

**Figure 8:**
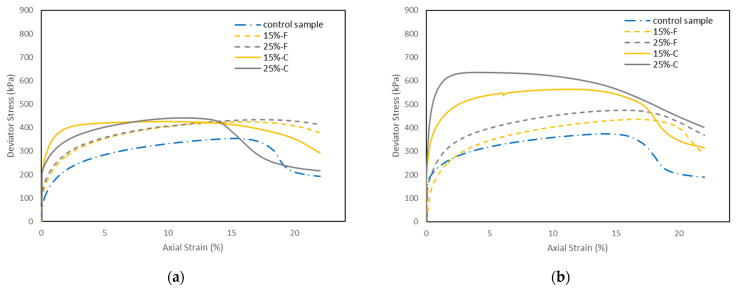

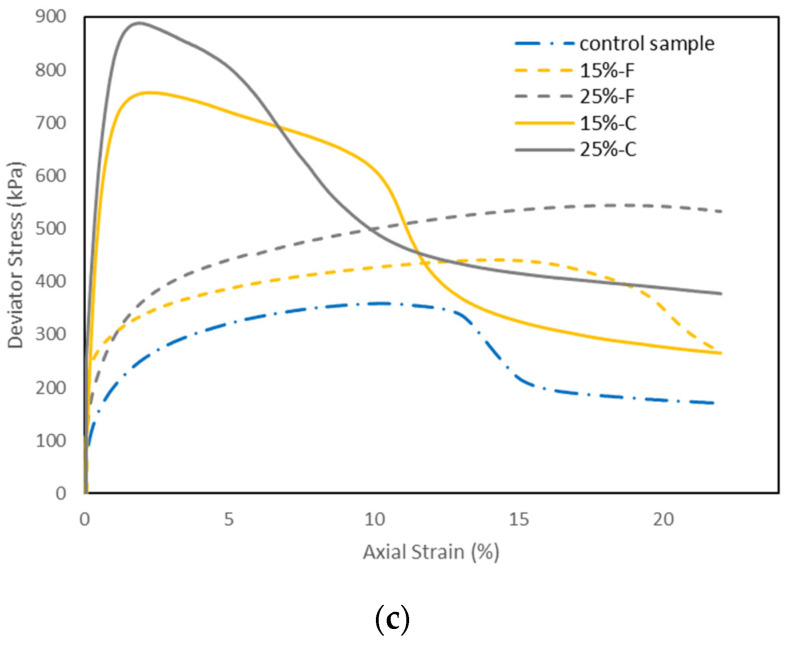
Stress-strain behavior of control sample and soil samples stabilized with class C and class F fly ash at a confining pressure of 600 kPa at (**a**) one day of curing, (**b**) seven days of curing, (**c**) 28 days of curing.

Figure 8a–c shows the deviator stress (q)—axial strain (ε_a_) behavior of the control sample and the soil samples stabilized with 15% and 25% class C and class F fly ash, cured for 1, 7, and 28 days, at 600 kPa effective confining pressure (σ_c_’). It is seen that at a given axial strain, the deviator stress of the soil stabilized with both types of fly ash increased with increasing fly ash content. This trend is consistent with the findings from Prabakar et al. [8]. However, a smaller improvement was observed with the class F fly ash compared to the class C fly ash. For both types of fly ash stabilized soil, the deviator stress increased by increasing the curing time. In addition, there was a significant increase in the deviator stress at 28 days of curing for the soils stabilized with class C fly ash, and the stress-strain curve showed a brittle post-peak strain-softening response (Figure 8c) compared to the generally observed postyield ductile behavior in soils stabilized with class C fly ash at one day and seven days of curing (Figure 8a,b). The results also show that for the soil stabilized with class C fly ash with 28 days of curing, the axial strain corresponding to the peak deviator stress decreased. For example, in the control sample the peak deviator stress (q_max_) reached 359 kPa at around 11% axial strain, while in the soil stabilized with 25% class C fly ash, the sample reached the q_max_ of 889 kPa at around 2% axial strain. This could be that the cementitious properties of class C fly ash produced stiff bridges in the soil structure, and lower strains are enough to break such bridges.

**Figure 9:**
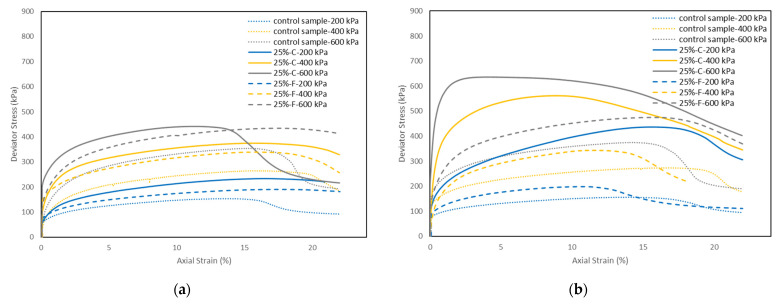

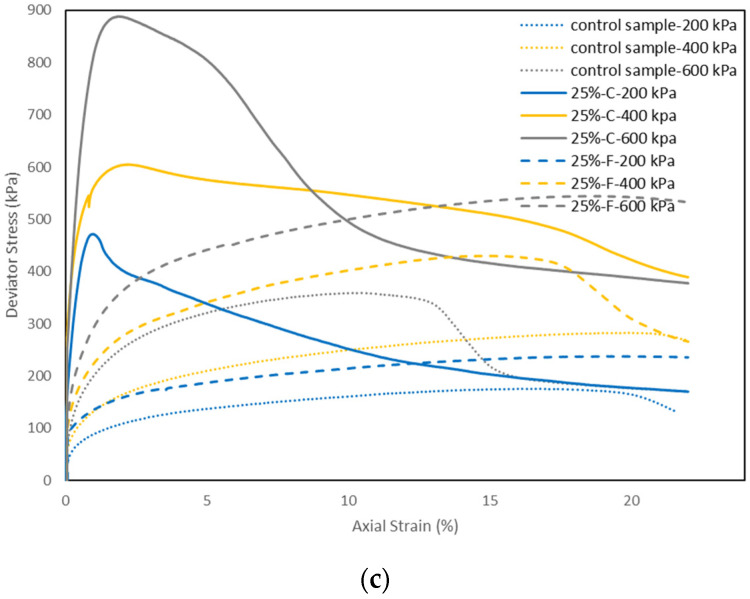
Stress-strain behavior of control sample and soil samples stabilized with 25% of class C and class F fly ash at confining pressures of 200, 400, and 600 kPa at (**a**) one day of curing, (**b**) seven days of curing, (**c**) 28 days of curing.

Figure 9a–c shows the deviator stress-axial strain behavior of the control samples, and the soil samples stabilized with 25% class C and class F fly ash at 200, 400, and 600 kPa confining pressures, cured for 1, 7, and 28 days, respectively. In general, the deviator stress of the soils stabilized with both types of fly ash increased with increasing the effective confining pressure at all curing times. This is because higher confining pressure during the consolidation stage decreases the void ratio, hence increasing the strength of the soil. The soil samples stabilized with class F fly ash generally showed a similar ductile stress-strain response with the increase of confining pressure. On the other hand, for the samples stabilized with class C fly ash at one and seven days of curing, the stress-strain response changed from ductile to slightly brittle strain-softening behavior with increasing the confining pressure (except for the sample tested at 400 kPa confining pressure and one day of curing). For the samples stabilized with class C fly ash at 28 days of curing, a brittle strain-softening behavior was observed for all confining pressures. However, the samples showed higher peak and became more brittle with increasing the confining pressure. The reason for the brittle strain-softening behavior is a slight destructuration that occurs in the class C fly ash stabilized soil during the consolidation stage, leading to the behavior governed by cementitious bonds and friction [34].

#### 5.3.2. Effects of Fly Ash Content on Shear Behavior of the Soil

The shear strength parameters of the soil were obtained from the Mohr–Coulomb failure criterion. Mohr circles were plotted for the control and stabilized soil samples at three different confining pressures.

The values of the effective angle of shearing resistance (ϕ’) and effective cohesion (c’) are shown in Table 4 for the control sample and the samples of soil stabilized with both types of fly ash at 1, 7, and 28 days of curing. The results indicate that the value of c’ increased with the addition of class C fly ash. These results agree with the observations made by other researchers [9,35]. For the soil stabilized with class C fly ash, the increase in c’ is significant with increase in curing times. On the other hand, the cohesion of the soil stabilized with class F fly ash is lower than the control sample at 1, 7, and 28 days of curing, although the cohesion of the stabilized soil samples increased with the curing times due to the pozzolanic reactions. This is because class F fly ash has silty characteristic and includes very low calcium reactive content. Hence, when the cohesionless fly ash mixes with the clay, the soil structure of the clay changes, leading to the decrease in cohesion. However, for the soil samples stabilized with class C fly ash, the chemical reaction between cementitious class C fly ash and clay has a significant effect in improving the cohesion even though the fly ash is cohesionless. Essentially, the reason for higher cohesion value in class C fly ash and lower value in class F fly ash could be the variation of the amounts of cementitious compound in the soils stabilized with two types of fly ash.

The values of the effective angle of shearing resistance improved with the addition of both types of fly ash. However, class C fly ash was more pronounced in comparison with class F fly ash in improving the effective angle of shearing resistance due to its chemical nature. According to Bryson et al. [36], the increase of ϕ’ is related to the particle substitution. The silty characteristic of fly ash decreased the clay fraction and increased the average grain size of the mixture. This contributed to improving the angle of shearing resistance. In addition, the ϕ’ value of the stabilized soil samples increased with the increase in curing time. This is because the self-hardening and pozzolanic properties of fly ash become more pronounced with curing. Sezer et al. [9] also indicated that the loss of moisture from a sample during curing may cause an increase in the value of ϕ’.

**Figure 10:**
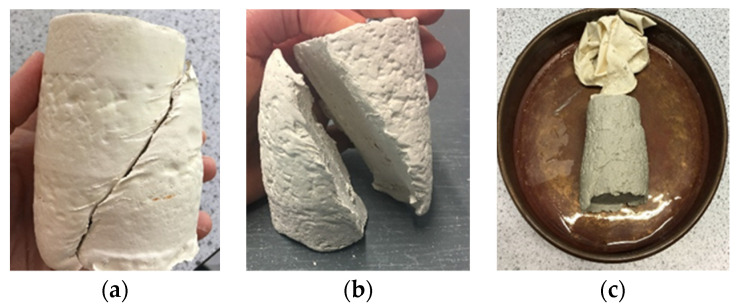
Sheared triaxial samples of: (**a**) the pure clay; (**b**) 15% fly ash-stabilized clay; (**c**) 25% fly ash-stabilized clay.

Figure 10a–c shows the control sample and the fly ash stabilized soil samples with clear shear failure through the samples. The samples reached critical state during the shearing stage of the triaxial tests.

#### 5.3.3. Effects of Fly Ash Content on Critical State Parameters

**Figure 11:**
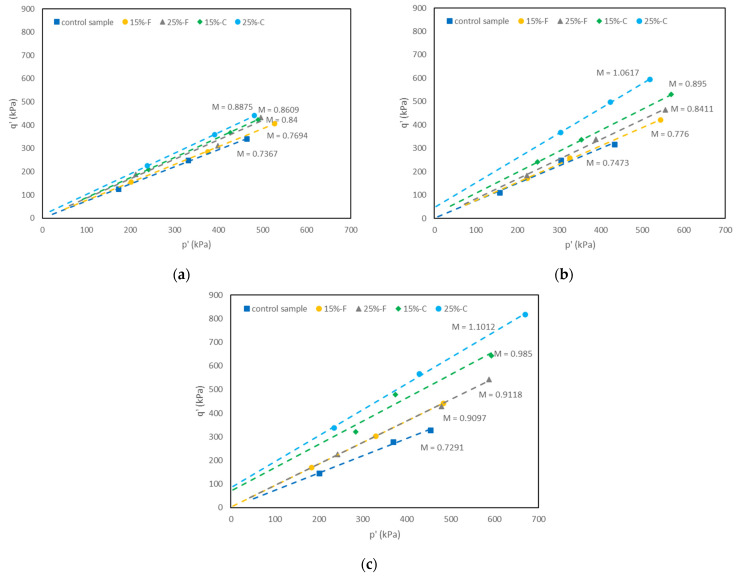
Critical state lines for the control sample and the soil samples stabilized with class C and class F fly ash at: (**a**) one day of curing, (**b**) seven days of curing, (**c**) 28 days of curing.

Figure 11a–c shows the critical state lines (CSL) of the control sample and the samples of the soil stabilized with 15% and 25% of class C and class F fly ash cured for 1, 7, and 28 days. Based on the results, the gradient of the CSL (M) increased with increasing the fly ash content for both types of fly ash and with increasing the curing time. There was a greater improvement in the value of M for the soil stabilized with class C fly ash in comparison with the soil stabilized with class F fly ash for all curing times. The parameter M is directly related to the angle of shearing resistance [37] and indicates the relationship between soil particles and their geometry. It can be said that the increase in angle of shearing resistance with class C fly ash content is because a certain amount of cement is necessary to react with the clay, hence, higher improvement in the value of M was observed with the class C fly ash content and lower improvement with the class F fly ash. Subramaniam et al. [38] reported similar observations for M value for a clay soil stabilized with low cement content.

The results also indicate that the y-intercept of the critical state lines in the space of deviator stress versus mean effective stress, increased with the addition of class C fly ash for all curing times (except the soil sample stabilized with 15% class C fly ash at one day curing). The y-intercept of the CSL is related to cohesion and is a result of cementation [39]. It could be concluded that class C fly ash gave high reaction due to the higher calcium content.

### 5.4. One-Dimensional Consolidation Tests

One-dimensional consolidation tests were carried out to evaluate the effects of fly ash on the consolidation characteristics of the soil including compression index (*C_c_*), swelling index (*C_s_*), volume compressibility (*m_v_*), coefficient of consolidation (c_v_), and permeability (*k*).

The compression index is one of the important parameters to determine the consolidation settlement of soft ground. Table 5 shows the variation of values of *C_c_* and *C_s_* with different fly ash contents and curing times. The results show that the value of *C_c_* of the soil decreased with increasing the class C fly ash content. This is consistent with the results reported in the literature [28,36,40]. This is because after the cation exchange reaction between class C fly ash and clay, the flocculation and aggregation of the soil increase. This improves the vertical effective yield stress and reduces the compressibility of the soil [41]. In this way, the addition of fly ash decreases the settlement of the soil. However, the compression index of the soil stabilized with 15% class F fly ash (at one day of curing) increased (compared with the control sample) and then showed a decrease with increasing the fly ash content to 25%.

In addition, the compression index decreased with the increase of curing time for both types of fly ash. This could be attributed to the pozzolanic characteristics of fly ash. When the pozzolanic reaction starts, cementitious particles gradually fill and reinforce the interparticle voids. Thus, the stabilized soil would be less compressible as it cures [42].

The results also indicate that the addition of fly ash and the increase of curing time reduced the *C_s_* of the soil (Table 5). This trend agrees well with the findings of Kolay and Ramesh [28], Bryson et al. [36], and Amiralian et al. [40]. According to Prabakar et al. [8], the nonexpansive characteristics of fly ash, and the shape and size of the particles of fly ash lead to the decrease of swelling characteristics.

The results in Table 6 also show that that class C fly ash had a better contribution to decreasing of the compression and swelling indices of the soil compared to class F fly ash.

The coefficient of volume compressibility (*m_v_*) represents the amount of change in unit volume due to a unit change in effective stress:

(1) $$m_{v}=\frac{\Delta e}{\Delta \sigma \prime }\times \frac{1}{1+e_{0}}$$

where Δ*e*/Δ*σ*′ is the slope of the *e*/*σ*′ curve [43].

**Figure 12:**
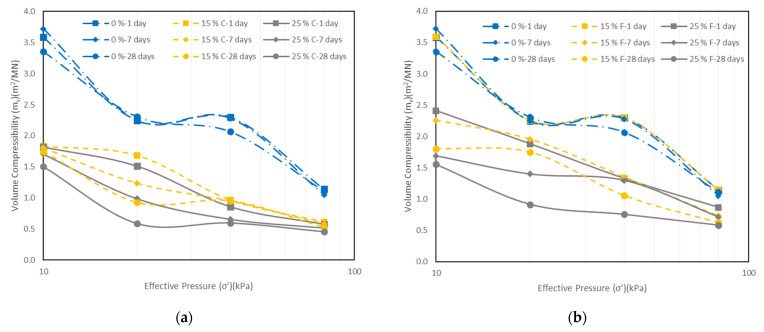
Variation of coefficient of *m_v_* with effective stress (*σ’*) for: (**a**) different class C fly ash contents, (**b**) different class F fly ash contents, and curing times.

**Figure 13:**
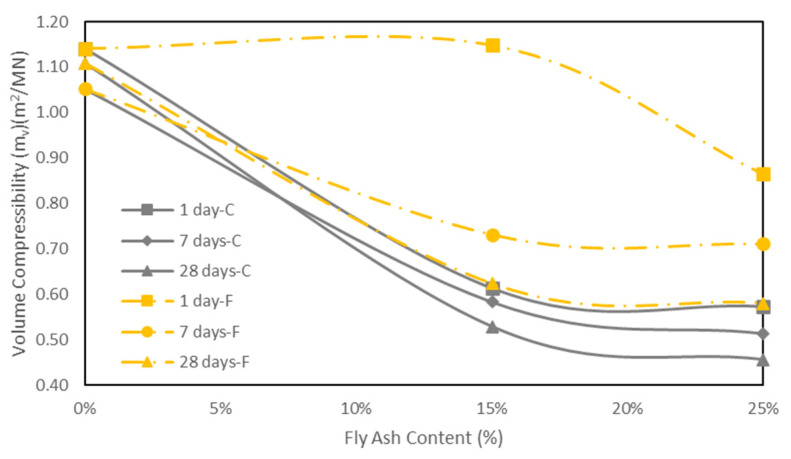
Variation of coefficient of *m_v_* with different fly ash contents at effective pressure of 80 kPa and different curing times.

The variations of the coefficient of *m_v_* with different fly ash contents and curing times are illustrated in Figure 12a,b and Figure 13. Figure 12a,b shows the changes in volume compressibility with the applied effective stress for the control sample and the soil samples stabilized with fly ash at 1, 7, and 28 days of curing. Figure 13 shows the variation of volume compressibility with the fly ash content for an effective pressure of 80 kPa at different curing times. The results show that for both types of fly ash the value of *m_v_* decreased with increasing fly ash content and curing days. For an effective stress of 80 kPa, the values of *m_v_* for the control sample were 1.14, 1.05, and 1.11 m^2^/MN at 1, 7, and 28 days of curing, respectively. For the soil stabilized with class C fly ash, the results indicated a decrease of 0.61, 0.58 and 0.53 m^2^/MN for 15% fly ash, and 0.57, 0.51, and 0.46 m^2^/MN for 25% fly ash at 1, 7, and 28 days of curing, respectively. However, for the soil stabilized with class F fly ash, the *m_v_* value slightly increased from 1.14 m^2^/MN to 1.15 m^2^/MN when fly ash content was increased from 0% to 15% at one day of curing. Thereafter, the values of *m_v_* decreased to 0.73 and 0.62 m^2^/MN for 15% fly ash at 7 and 28 days, and 0.86, 0.71 and 0.58 m^2^/MN for 25% fly ash at 1, 7 and 28 days of curing, respectively (Figure 13).

The coefficients of consolidation of the control samples and fly ash stabilized samples at different curing days were estimated based on Taylor’s square root of time method [40].

The coefficient of permeability (k) of the samples was also estimated based on the results of the coefficient of consolidation (c_v_), the coefficient of volume compressibility (m_v_), and the unit weight of water (γ_w_) [43]:

K = c_v_*m_v_*γ_w_ (2)

Table 6 shows the variations of c_v_ and k for different fly ash contents and curing times for an effective stress increment of 40–80 kPa. The results show that the value of c_v_ of the soil increased with the inclusion of fly ash. A similar trend was observed from the analysis of permeability results. According to Wang and Tanttu [44], the increase in c_v_ leads to an increase in permeability. The permeability of the soil increased with the increase of fly ash content. This trend is comparable with the finding of Phanikumar [45]. According to Chew et al. [42], the Ca^2+^ ions from the fly ash lead to the formation of a flocculated structure in the clay. The flocculation leads to increase of permeability [45]. Mir and Sridharan [46] also indicated that the soil with inclusion of fly ash becomes coarser in comparison with the control sample. In this way, the stabilization of soil with fly ash increases the permeability of the soil. Jaditager and Sivakugan [47] reported that the permeability of dredged mud increased with fly ash based geopolymer. They argued that, due to the improvement in the permeability, the pores and hole cavities in the stabilized samples allow easy drainage of water during the primary compression. However, an increase of curing time results in the decrease of c_v_ and k for the fly ash stabilized soil. The soil stabilized with class C fly ash showed a higher permeability than the control samples with one day and seven days of curing. However, the stabilized soil became less permeable in comparison with the control sample at 28 days of curing. On the other hand, the class F fly ash stabilized soils showed a higher k value than the control samples with 1, 7, and 28 days of curing, although the k value of the stabilized samples decreased with the curing time. The long-term decrease of permeability is due to the reaction of calcium aluminium silicate hydrate (CASH) and/or calcium silicate hydrate (CSH) [42]. This cementitious gel is deposited in the pores of stabilized soil during curing [48]. Chew et al. [39] analyzed cement treated clays and found that the permeability of soil increased with an increase of cement content, and the permeability of cement stabilized soil decreased with time. Kassim and Chow [48] reported a similar trend by testing a clay soil stabilized with lime. They argued that high permeability in early stages of curing and a decrease in permeability during curing can bring an advantage in geotechnical applications.

### 5.5. Scanning Electron Microscopy

**Figure 14:**
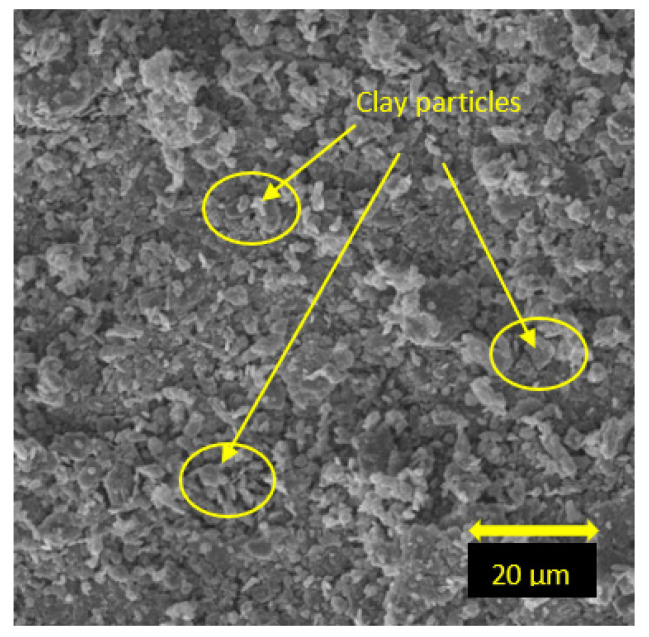
SEM images of clay (control sample).

The SEM analysis was carried out to evaluate the microstructural changes in the soil with the increase of curing days and fly ash percentages. Figure 14 shows the microstructure of the unstabilized soil. The image shows the plate-like clay particles with dense and regular fabric which was also reported by Jaditager and Sivakugan [49].

**Figure 15:**
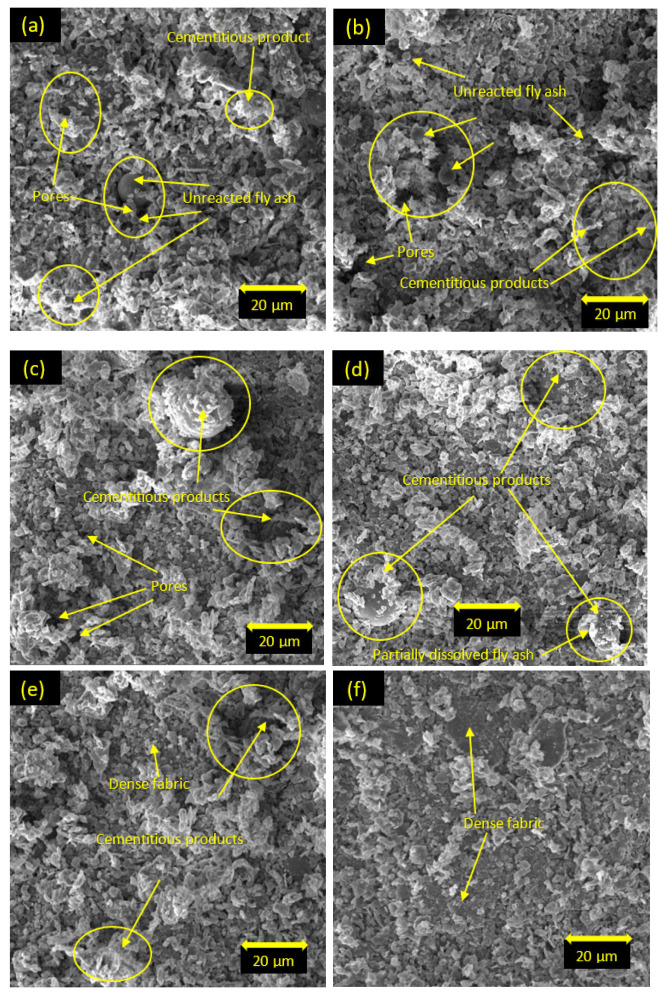
SEM images of soil stabilized with class C fly ash: (**a**) 15% C, one day of curing; (**b**) 25% C, one day of curing; (**c**) 15% C, seven days of curing; (**d**) 25% C, seven days of curing; (**e**) 15% C, 28 days of curing; (**f**) 25% C, 28 days of curing.

**Figure 16:**
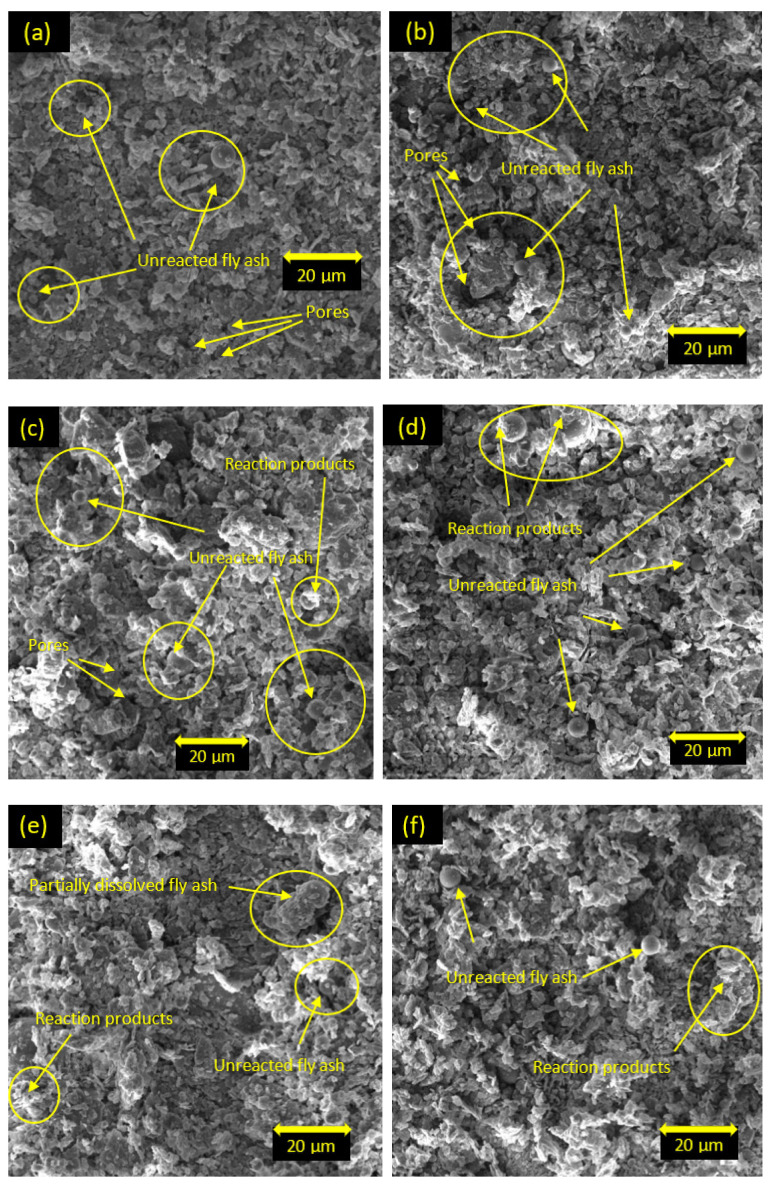
SEM images of soil stabilized with class F fly ash: (**a**) 15% F, one day of curing; (**b**) 25% F, one day of curing; (**c**) 15% F, seven days of curing; (**d**) 25% F, seven days of curing; (**e**) 15% F, 28 days of curing; (**f**) 25% F, 28 days of curing.

The microstructures of the soil stabilized with class C and class F fly ash are shown in Figure 15 and Figure 16, respectively. For one day of curing, the microstructures of the soil stabilized with both class C and class F fly ash (Figure 15a,b and Figure 16a,b) contained scattered and aggregated clay particles, spherical-unreacted fly ash particles, and pores with hollow cavities. The morphology of the soil stabilized with class C fly ash at one day of curing also showed the presence of C-S-H cementitious products around the fly ash particles. For seven days of curing, more cementitious products were formed and bonded around fly ash particles for the soil stabilized with class C fly ash (see Figure 15c,d). The soil stabilized with 25% class C fly ash (Figure 15d) showed more cementitious products compared to the soil stabilized with 15% of class C fly ash (Figure 15c). For 28 days of curing, class C fly ash particles were covered with the cementitious products, and the products also filled the pore spaces and improved the interlocking structures which contributed to the dense fabric in the stabilized soil (Figure 15e,f). In addition, the structure of the soil presented a denser fabric, and no unreacted fly ash was observed with the increase of fly ash content and curing time (Figure 15f). The denser fabric could result in higher strength and stiffness in the stabilized soil [33,50,51,52,53]. Therefore, the results of the UCS, triaxial tests, and oedometer tests in this study are consistent with the SEM observations. The results of SEM analysis for the soil stabilized with class F fly ash are similar to those for the soil stabilized with class C fly ash. However, the morphology of soil stabilized with class F fly ash presented more unreacted fly ash and less reaction products due to the lack of Ca ions (Figure 16). For 7 and 28 days of curing, the soil stabilized with class F fly ash showed the presence of reaction products due to the pozzolanic reactions, and therefore the structural bonding of the stabilized soil was improved. However, unreacted fly ash and partially dissolved fly ash particles were still observed in the stabilized soil. It can be said that the microstructure of the soil stabilized with class F fly ash was modified insignificantly with the increase of curing times. As a result, the soil stabilized with class F fly ash indicated lower strength compared to the soil stabilized with class C fly ash.

## 6. Conclusions

This paper investigated the influence of class C and class F fly ash on the strength, consolidation, and microstructural characteristics of a clay soil. The following conclusions can be drawn from the results presented in this paper.

- The maximum dry unit weight of the soil decreased, and the optimum moisture content increased with increasing percentages of both types of fly ash. The soil stabilized with class C fly ash had higher γ_dmax_ and lower w_opt_ in comparison with the soil stabilized with class F fly ash.
- The compressive strength of the soil increased with the addition of both types of fly ash and with the curing time. However, when the fly ash content increased from 25% to 30%, the compressive strength of the stabilized soil slightly decreased for both types of fly ash and for different curing times. Therefore, the optimal fly ash content appears to be 25% for both types of fly ash. Also, class C fly ash was found to be much more effective in improving the compressive strength of the soil than class F fly ash. The elastic modulus of the soil increased with the addition of both types of fly ash up to 25% and with increasing the curing time.
- The results of the CU triaxial tests indicated an improvement in the angle of shearing resistance and cohesion intercept with the addition of class C fly ash, whereas the cohesion intercept of the soil stabilized with class F fly ash was lower than the control sample. The curing time was effective in improving the values of c’ and ϕ’ for both types of fly ash stabilized soil. The gradient of the critical state line increased with increasing the class C and class F fly ash contents and with increasing the curing time.
- The results from the one-dimensional consolidation tests indicated a decrease in the C_c_ of the soil stabilized with class C fly ash compared to the control sample. Furthermore, the Cc decreased with curing time for both types of fly ash. However, with class F fly ash, the Cc initially increased up to the particular fly ash content and thereafter decreased at one day of curing. The mv value showed a similar trend to the compression index. Cs decreased by the addition of the class C or class F fly ash. In addition, curing time was found to be an effective parameter in decreasing the swelling index.
- The values of cv and k increased with the addition of class C or class F fly ash. However, both cv and k decreased with increasing the curing time for fly ash stabilized soils.
- The SEM analysis conducted on the soil stabilized with both class C and class F fly ash confirmed the gradual improvement in the soil properties and strength due to the formation of reaction products in the soil with the increase of curing time. However, the soil stabilized with class C fly ash had more reaction products and denser fabric than the soil stabilized with class F fly ash due to the better cementitious properties of class C fly ash. The results from the UCS, triaxial, and consolidation tests were found in agreement with the results from the SEM analysis.

In general, it was observed that class C fly ash is more effective in improving the mechanical properties of the soil compared to class F fly ash. The findings have proven that class C fly ash can be used effectively in the stabilization of clay soils. Class F fly ash can be used with the other additives such as lime or alkali activators to achieve higher mechanical properties in clay soils.

## Figures and Tables

**Table 1:** Summary of available literature on fly ash stabilized clay soil.

Reference	Type of Soil	Stabilization Agent	Test Carried Out	Main Results
Cokca [7]	Expansive soil	High calcium and low calcium fly ash (0–25%)	Atterberg limits, swelling potential	The classification of high calcium (25%) and low calcium (25%) fly ash changed from CH to ML, and CL, respectively, 65% and 68% decrease in swelling potential with high calcium and low calcium fly ash (25% content), respectively
Prabakar et al. [8]	CL, OL, MH	Fly ash (9–46%)	compaction, shear strength, free swell, CBR	15–20% dry density reduction, nonlinear increase of shear strength, decrease of swelling behavior, increase in CBR value
Sezer et al. [9]	CH	High lime fly ash (0–15%)	Compaction, UCS, shear strength	Decrease in MDD and increase in OMC, improvement in cohesion, angle of friction, and UCS
Phanikumar and Sharma [10]	CH	Low calcium fly ash (0–20%)	Plasticity, shear strength, swelling, compaction	About 50% decrease in swelling potential and plasticity index, 27% increase in undrained shear strength, 25% decrease in OMC, 5% increase in MDD with 20% fly ash content
Senol et al. [11]	Clay	Class C fly ash (10–20%)	Compaction, UCS, CBR	Decrease in MDD, increase in OMC, increase in CBR values and UCS
Edil et al. [12]	CL, CH, OH	Class C fly ash (0–30%)	CBR	4 and 8 times increase in CBR values with 10% and 18% fly ash content, respectively on CL and CH, insignificant improvement on OH
Phanikumar and Sharma [13]	CH	Class C fly ash (0–20%)	Oedometer, free swell	About 50% decrease in free swell index, significant decrease in compression index
Bin-Shafique et al. [14]	CH, CL	Class C fly ash (0–20%)	Plasticity index, UCS, and vertical swell test with wet-dry cycles and freeze-thaw cycles	4 times increase in UCS on both soil types, about 75% decrease in swelling potential, about 50% decrease in plasticity with 20% fly ash content, no significant effect on test parameters with wet-dry cycles, small decrease in UCS with freeze-thaw cycles
Brooks [15]	CH	Class C fly ash (0–25%)	Swell-shrinkage, UCS	About 106% and 50% increase in failure stress and strain, respectively with 25% fly ash content, and decrease in swelling potential
Tastan et al. [16]	Organic clay	Class C and F fly ash (10–30%)	UCS	Increase in UCS with a decrease of organic content of soil, and an increase of Ca amount of fly ash
Seyrek [17]	CH, CL	Class C and F fly ash (0–30%)	Atterberg limits, swell pressure, UCS, compaction	Decrease in plasticity index up to addition of 20% of fly ash, decrease in swelling potential and increase in UCS up to 25% fly ash content, decrease in MDD, increase in OMC
Jose et al. [18]	Expansive soil	Class F fly ash (0–15%)	Atterberg limit, Free swell, UCS	About 36% decrease in liquid limit, 43% increase in compressive strength, and decrease in free swell index from 71% to 39% with 15% fly ash addition

**Table 2:** Oxide composition of fly ashes.

Chemical Composition	Class C Fly Ash	Class F Fly Ash
SiO_2_ (%)	28.3	48.6
CaO (%)	32.4	2.2
Fe_2_O_3_ (%)	6.6	9.2
Al_2_O_3_ (%)	15.8	22.5
K_2_O (%)	0.5	4.1
MgO (%)	4.2	1.3
Na_2_O (%)	0.3	0.9
P_2_O_5_ (%)	0.7	0.2
SO_3_ (%)	8.6	0.9
TiO_2_ (%)	0.9	1.1

**Table 3:** Elastic modulus of class C and class F fly ash stabilized soil from UCS tests with different curing times.

	1 Day Curing	7 Days Curing	28 Days Curing
Fly Ash Content	Elastic Modulus (E) (MPa)		
0% (control sample)	5.9	9.5	9.4
5% class C	16.5	21.3	23.1
10% class C	20.2	25.9	39.1
15% class C	25.2	33.7	42.6
20% class C	25.4	51.0	62.9
25% class C	26.2	53.6	64.9
30% class C	27.5	35.0	37.5
5% class F	8.8	12.5	14.7
10% class F	9.0	13.5	18.2
15% class F	14.0	14.1	21.0
20% class F	14.8	16.8	21.3
25% class F	18.5	21.2	24.8
30% class F	14.8	9.0	14.6

**Table 4:** Shear strength parameters of control and fly ash stabilized soil samples with different curing times.

Fly Ash Content (%)	Curing (Days)	c’ (kPa)	φ’ (Deg.)
0%	1	17.5	18.1
0%	7	18.5	19.6
0%	28	19.0	18.4
15% class C	1	15.6	21.4
15% class C	7	43.0	21.8
15% class C	28	86.6	22.5
25% class C	1	20.2	21.8
25% class C	7	77.8	23.0
25% class C	28	99.1	24.0
15% class F	1	2.7	20.7
15% class F	7	4.9	21.1
15% class F	28	11.1	22.3
25% class F	1	8.4	21.6
25% class F	7	10.1	21.6
25% class F	28	15.1	22.4

**Table 5:** Effects of fly ash and curing time on compression and swelling indices.

Fly Ash Content (%)	Curing (Days)	Compression Index (*C_c_*)	Swelling Index (*C_s_*)
0% (control sample)	1	0.277	0.054
0% (control sample)	7	0.256	0.046
0% (control sample)	28	0.270	0.046
15% class C	1	0.164	0.038
15% class C	7	0.156	0.022
15% class C	28	0.140	0.015
25% class C	1	0.154	0.037
25% class C	7	0.139	0.021
25% class C	28	0.123	0.015
15% class F	1	0.288	0.046
15% class F	7	0.187	0.043
15% class F	28	0.161	0.037
25% class F	1	0.227	0.045
25% class F	7	0.185	0.043
25% class F	28	0.153	0.029

**Table 6:** Effects of fly ash and curing time on coefficient of consolidation and permeability.

Fly Ash Content (%)	Curing (Days)	Coefficient of Consolidation (C_v_) (mm^2^/min)	Permeability (k) (m/min)
0% (control sample)	1	1.9	2.2 × 10^−8^
0% (control sample)	7	2.3	2.3 × 10^−8^
0% (control sample)	28	2.1	2.2 × 10^−8^
15% class C	1	8.2	5.0 × 10^−8^
15% class C	7	5.5	3.2 × 10^−8^
15% class C	28	4.2	2.2 × 10^−8^
25% class C	1	9.4	5.3 × 10^−8^
25% class C	7	5.5	2.8 × 10^−8^
25% class C	28	4.0	1.8 × 10^−8^
15% class F	1	6.3	7.1 × 10^−8^
15% class F	7	5.7	4.1 × 10^−8^
15% class F	28	5.3	3.2 × 10^−8^
25% class F	1	8.7	7.4 × 10^−8^
25% class F	7	6.0	4.2 × 10^−8^
25% class F	28	5.8	3.3 × 10^−8^

## Data Availability

Some of the data used to support the findings of this study can be made available from the corresponding author upon request.
